# Neuromonitoring in Neonatal-Onset Epileptic Encephalopathies

**DOI:** 10.3389/fneur.2021.623625

**Published:** 2021-02-02

**Authors:** Regina Trollmann

**Affiliations:** Department of Pediatrics and Pediatric Neurology, Friedrich-Alexander-Universität Erlangen-Nürnberg, Erlangen, Germany

**Keywords:** neonatal brain, genetic epilepsy, electroencephalopgraphy, metabolic epilepsy, suppression burst

## Abstract

Considering the wide spectrum of etiologies of neonatal-onset epileptic encephalopathies (EE) and their unfavorable consequences for neurodevelopmental prognoses, neuromonitoring at-risk neonates is increasingly important. EEG is highly sensitive for early identification of electrographic seizures and abnormal background activity. Amplitude-integrated EEG (aEEG) is recommended as a useful bedside monitoring method but as a complementary tool because of methodical limitations. It is of special significance in monitoring neonates with acute symptomatic as well as structural, metabolic and genetic neonatal-onset EE, being at high risk of electrographic-only and prolonged seizures. EEG/aEEG monitoring is established as an adjunctive tool to confirm perinatal hypoxic-ischemic encephalopathy (HIE). In neonates with HIE undergoing therapeutic hypothermia, burst suppression pattern is associated with good outcomes in about 40% of the patients. The prognostic specificity of EEG/aEEG is lower compared to cMRI. As infants with HIE may develop seizures after cessation of hypothermia, recording for at least 24 h after the last seizure is recommended. Progress in the identification of genetic etiology of neonatal EE constantly increases. However, presently, no specific EEG changes indicative of a genetic variant have been characterized, except for individual variants associated with typical EEG patterns (e.g., *KCNQ2, KCNT1*). Long-term monitoring studies are necessary to define and classify electro-clinical patterns of neonatal-onset EE.

## Introduction

Seizures are the most common clinical manifestation of central nervous system dysfunctions in newborns, with a reported incidence of up to 130/1,000 very low birth weight infants (birth weight lower than 1,500 g) and 1.5–3.5/1,000 term newborns (1–3). The leading causes of neonatal seizures in pre-term and term newborns are acute symptomatic etiologies, such as perinatally acquired hypoxic-ischemic (HI), haemorrhagic and inflammatory or infectious brain injuries, including unfavorable higher-grade intraventricular hemorrhage in pre-terms, perinatal HI encephalopathy (HIE), thrombo-embolic brain injury, and perinatal stroke (1, 4–8).

Etiologically more variable and especially challenging is the group of early-onset epileptic encephalopathies (EOEE) including neonatal-onset epileptic encephalopathies (EE) that are mainly of structural, metabolic and genetic etiology. Early-onset epileptic encephalopathies have been defined by the ILAE as the presence of frequent epileptiform activity that is usually associated with high seizure frequency, a primary pharmaco-refractory course and global impairment of development (9). Along with the new ILAE definition of developmental and epileptic encephalopathies (9), the current ILAE Neonatal Task Force guidelines (2017) primarily include age-specific etiologies and comorbidities in the recently revised classification of neonatal epilepsies. Early-onset epileptic encephalopathies with burst suppression represent a group of the most severe neonatal-onset EE including Ohtahara syndrome (OS), often associated with structural brain anomalies, and early myoclonic encephalopathy (EME), typically associated with inherited metabolic disorders; however, recent advances in genetics have elucidated a wide range of pathogenic genetic variants in these age-dependent EE (10), as well as severe neonatal-onset EE other than OS and EME (11–13).

Clinical studies have indicated the prognostic significance of prolonged neonatal seizures, including non-convulsive and subclinical seizures, in pre-term and critically ill neonates. They may increase the risk of intellectual disability, epilepsy, and infantile cerebral palsy (3, 14–18). In a group of 76 pre-term newborns with neonatal seizures, Pisani et al. (3) found a significant correlation of birth weight, seizure onset, neurologic examination and EEG with neurodevelopmental outcomes at 1 year of age in neonates with status epilepticus compared to the pre-term newborns with seizures but without status. All infants with status epilepticus had an unfavorable outcome compared to only 22.3% of those with neonatal seizures. Moreover, comprehensive experimental studies have supported the hypothesis that neonatal seizures exert various and long-lasting effects on brain maturation and plasticity as well as permanent reduction of the seizure threshold. Profound dysregulations of neuronal and glial proliferation and migration, as well as disturbed synaptogenesis and maturation of neurotransmitter systems such as GABAergic synaptic transmission in response to experimental seizures, are suggested as main contributing factors to the high sensitivity of the immature brain to seizures (19–21). This highlights the need for early identification of clinical and electrographic-only seizures as well as for reliable therapy monitoring.

According to the American Clinical Neurophysiology Society Guideline 2011 (22), EEG is highly sensitive and essential for diagnosing and monitoring neonatal seizures and EOEE. However, age-specific semiology and variability in the EEG maturational pattern and electro-clinical dissociation make interpreting diagnostic and prognostic EEG parameters in very pre-term and critically ill neonates particularly challenging (3, 8, 23, 24). Moreover, limited data are available on electro-clinical phenotypes of rare genetic neonatal-onset EEs, and specific EEG changes suggestive of a causative variant have not been characterized. This article reviews the significance and challenges of conventional and amplitude-integrated EEG (aEEG) monitoring in neonatal-onset EE with a special focus on acute symptomatic as well as specified rare genetic neonatal-onset EE.

## Neonatal EEG

Multichannel video-EEG (vEEG) is recommended as the gold standard for monitoring neonates with an increased risk of neonatal encephalopathy and/or seizures, and continuation of multichannel vEEG monitoring is proposed for at least 24 h after the last electrographic seizure (22). For continuous neuromonitoring in high-risk newborns (Table 1), aEEG is well-established in many neonatal intensive care units and considered a valuable bedside monitoring tool [for a review, see Hellström-Westas (25)], especially for seizure detection and treatment monitoring (16, 26–31).

**Table 1:** Recommended indications for aEEG monitoring in preterm and term newborns according to the American Clinical Neurophysiology Society's Guideline on continuous electroencephalography monitoring in neonates [Shellhaas et al. (22)].

**Age-group**	**Indication**
Term	Perinatal Hypoxic-ischemic encephalopathy (HIE)
Preterm/term	Infection, sepsis, meningitis
Preterm/term	Encephalopathy of metabolic and genetic origin
Preterm/term	Intracranial hemorrhage
	Perinatal/neonatal stroke
Preterm/term	Neonatal seizures (diagnosis, treatment monitoring)
Preterm/term	Congenital heart disease, ECMO, interventions
Preterm/term	Anticonvulsive treatment (electro-clinical dissociation)
	Treatment with sedatives, anesthetics, analgesics, and paralytics

### Multichannel vEEG

Neonatal conventional EEG remains a highly sensitive neurophysiological method for detecting electrographic seizure activity and diagnosing and monitoring neonatal seizures of acute symptomatic etiology as well as neonatal EE of structural, metabolic or genetic origin. In addition, the detection of physiological paroxysmal transients defining electrographic brain maturation from the pre-term to term developmental stage is of prognostic value and the exclusive domain of conventional, multichannel EEG (22, 32).

**Figure 1:**
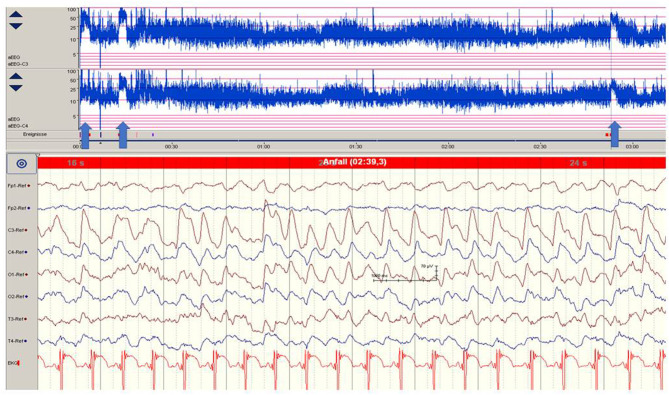
Focal epileptic discharges (left central region) in a 12-h-old neonate with perinatal stroke in the MCA territory. Saw-tooth pattern (arrows) of the aEEG (C3, C4) point to repetitive electrographic seizure activity. (Calibration is given in the figure, low-pass 30 Hz, high-pass 0.5 Hz).

**Figure 2:**
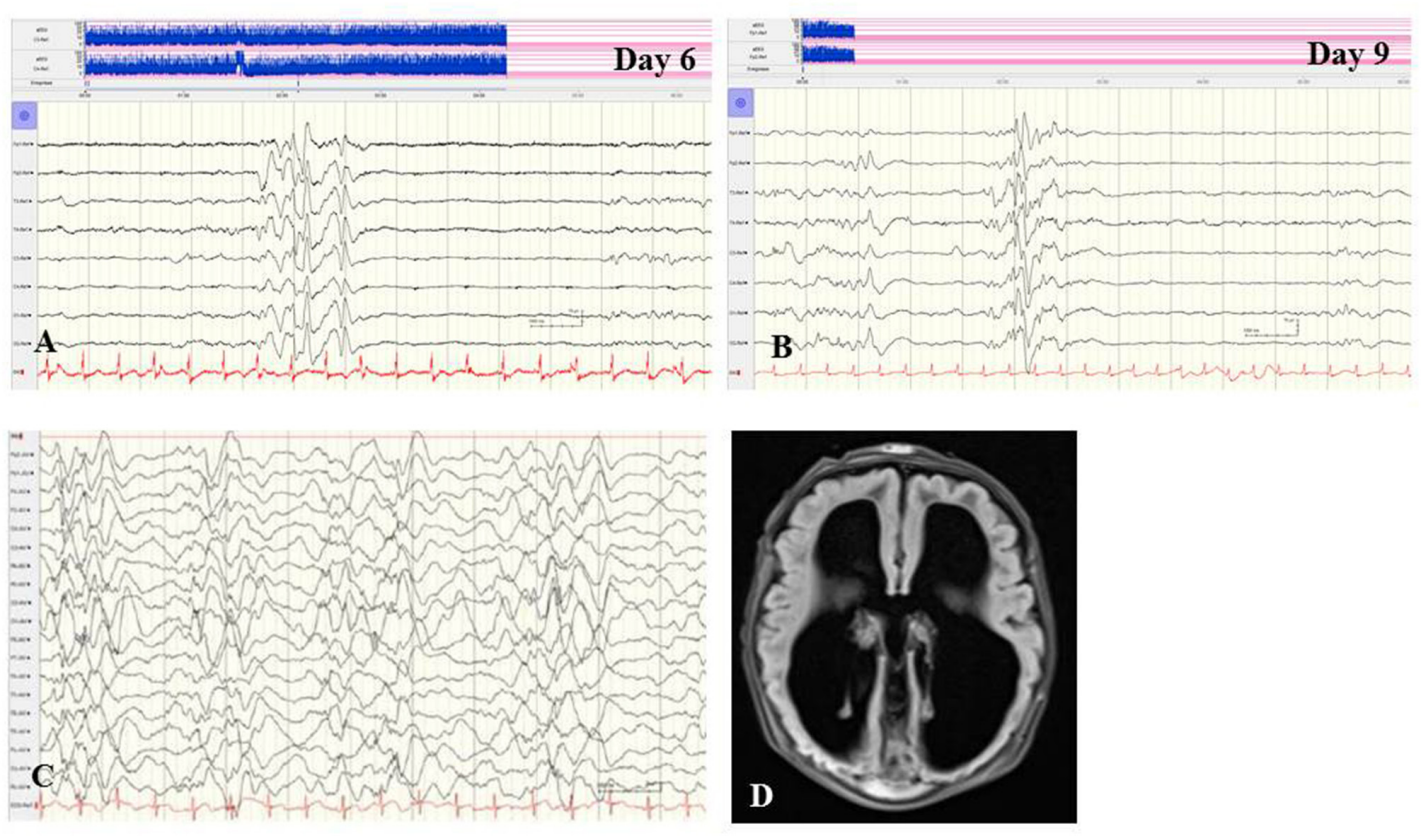
**(A,B)** EEG of a female term newborn with non-ketotic hyperglycinemia presenting apathy and hyperexcitability from day 1. First clinical seizures occurred at day 5. EEG at day 6 **(A)** and at day 9 **(B)** showed burst suppression with severe suppression (<4 μV) and intermittent high-voltage burst with diffuse spikes. **(C,D)** EEG and cMRI findings of a female neonate presenting myoclonic seizures, respiratory insufficiency, muscular hypotonia, hypoglycemia, and elevated CSF lactate and alanine levels. Interictal **(C)** and ictal EEG showed burst suppression during awake and asleep periods. **(D)** Brain MRI showed agenesis of corpus callosum and polymicrogyria. Heterozygous X-linked frameshift deletion in PDHα1 gene (skewed-X-chromosome inactivation) was detected. (Calibration is given in the figure, low-pass 30 Hz, high-pass 0.5 Hz).

Clinical diagnosis of seizures in neonates with paroxysmal motor or autonomic phenomena is associated with a high rate of misdiagnosis (33). Also, electro-clinical dissociation (uncoupling) is common in neonates with seizures and is pronounced in very pre-term and critically ill infants, in particular in neonates receiving sedative, paralytic, or anticonvulsive drugs (29–31, 34). Thus, as proposed by the American Clinical Neurophysiology Society, video-EEG according to the international 10–20 system of electrode placement, modified for neonates, is the gold standard (22, 32) to diagnose neonatal seizures based on ictal epileptic discharges. One must be aware of the variability of electrographic patterns and evolution of discharges in neonates mainly determined by maturational aspects (2, 22, 32). In neonates, epileptic sharp transients typically occur unilaterally repetitively or in series (2, 32, 35, 36), and ictal electrographic activity is characterized by paroxysmal, repetitive, evolving and stereotyped pattern and duration of 10 s or more (8). Also, brief rhythmic discharges (BRDs) of <10 s have been found in association with brain lesions and electrographic seizures in pre-term and term neonates (34, 35, 37, 38). The role of repetitive slow sharp wave activity (0.5–1 Hz) in extremely pre-term infants observed in association with intraventricular hemorrhages and increased mortality during the first postnatal days remains unclear (34, 39). Whereas, occipital onset is preponderate in very pre-term infants (27), onset in the central and temporal (GA > 32 weeks) and frontal regions (GA > 29 weeks) is found with increasing GA (27, 40–42) in patients with seizures of different etiology. Focal sharp waves with a normal background or ipsilateral suppression are found in neonates with perinatal stroke in the MCA territory (36) (Figure 1). These findings may be distinguished from focal epileptic discharges in non-lesional, genetic benign familial neonatal convulsions (BFNC) by background activity, being normal in BFNC. Ictal and interictal epileptic discharges associated with a severely pathological background pattern may indicate severe symptomatic or genetic EE. In term infants with acute brain insults (e.g., HIE), burst suppression patterns differs from burst suppression in neonates with neonatal-onset EIEE and EME. The latter (also called suppression burst) is characterized by high-amplitude bursts associated with diffuse epileptic discharges and fast activities, as well as intermittent periods of suppression (<10 s) (Figure 2A) that are typically shorter in EIEE/EME than in neonates with HIE and burst suppression (22, 32). In very pre-term infants with acquired acute brain injury, repetitive spikes, sharp waves and rhythmic sharp theta and delta transients associated with a suppressed background activity were more predictive of unfavorable outcomes than seizure duration (34). In general, the background EEG pattern is proposed as a useful prognostic marker in neonatal seizures and early-onset EE (13, 43, 44). However, its significance depends on the primary etiology and disease as well as modifying factors such as anticonvulsive, sedative and analgesic medications. For example, transient background suppression or increased discontinuity also have been found after bolus administration of phenobarbital or phenytoin (29–31) or surfactant application (45).

### Amplitude-Integrated EEG

**Figure 3:**
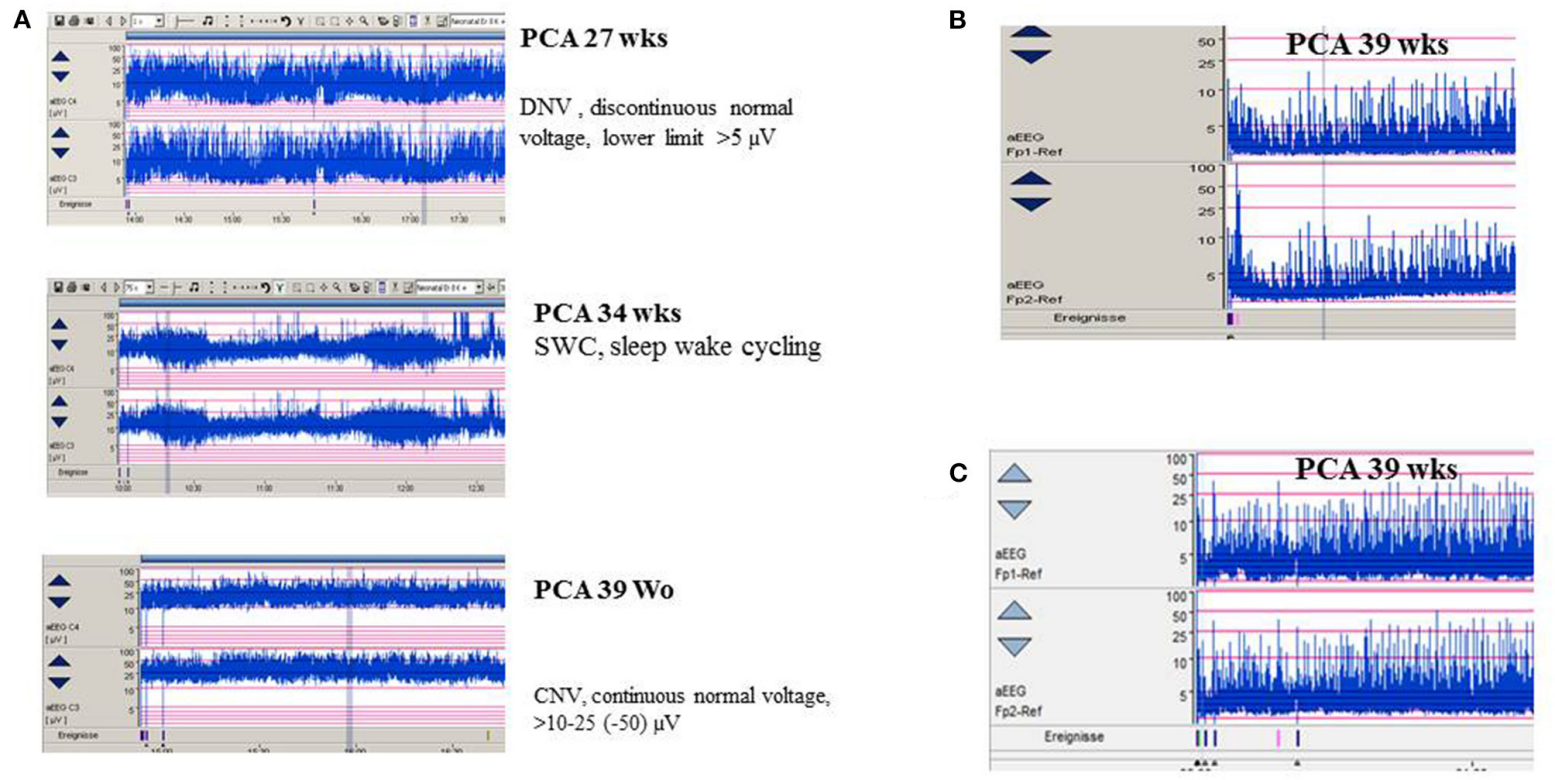
**(A)** aEEG findings of normal background activity in relation to maturational age. **(B,C)** Pathological background suppression classified according to Hellström-Westas (46). **(B)** PCA 39 weeks (postnatal age of 2 h), continuous extremely low voltage (CLV) tracing. **(C)** PCA 39 weeks (postnatal age of 5 h) burst suppression. (Calibration is given in the figure, low-pass 30 Hz, high-pass 0.5 Hz).

**Figure 4:**
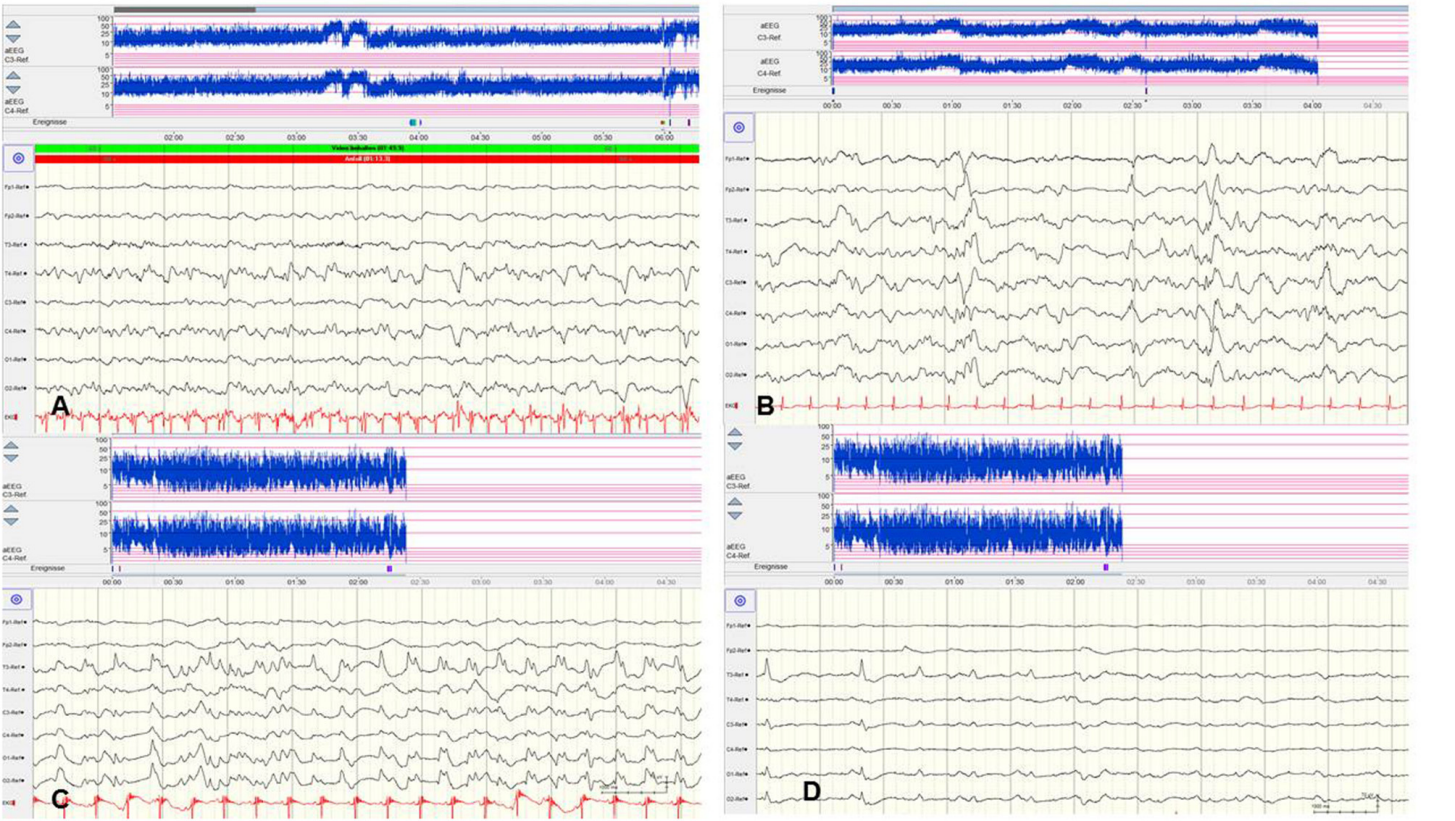
Focal epileptic discharges with a normal **(A,B)** and suppressed background **(C,D)**. **(A,B)** EEG in a 2-day-old term newborn presenting tonic seizures with opisthotonos and apnoea. Ictal EEG **(A)** showed focal epileptic discharges that were also present during interictal periods **(B)**. A saw-tooth pattern of the aEEG (C3, C4) indicates repetitive subclinical electrographic seizure activity. BFNC caused by a heterozygous KCNQ2 mutation was diagnosed. Neurodevelopmental outcome was normal. **(C,D)** EEG in a 10-h-old term newborn with moderate perinatal HIE (Sarnat stage II) and therapeutic hypothermia. Multichannel EEG **(C,D)** shows prolonged focal epileptic discharges and severely suppressed background activity. Suppressed lower amplitudes (<5 μV) and saw-tooth pattern of the aEEG (C3, C4) are shown. (Calibration is given in the figure, low-pass 30 Hz, high-pass 0.5 Hz).

Amplitude-integrated EEG (aEEG) is recommended as a useful bedside monitoring method, especially in neonates being at high risk of electrographic-only and prolonged seizures as well as status epilepticus (Table 1). Modern digital systems facilitate recording a multichannel vEEG using 6–8 (-10) active electrodes according to the international 10–20 system (including central and parietal electrode derivations), combined with the option that the aEEG trend that is given in a time-compressed and semilogarithmically transformed display is available for each channel [for a review see Hellström-Westas (25)]. Characteristic maturational aEEG background patterns are exemplarily demonstrated in Figure 3 [for a comprehensive review, see (46, 47)]. Recognition and interpretation of critical aEEG patterns by an experienced team of the neonatal intensive care unit without specific expertise in neurophysiological diagnostics is possible (25), such as the “saw-tooth pattern” of the aEEG trend that points to repetitive electrographic seizure activity (Figures 4A–D) or the typical pattern of background suppression (Figures 4C,D).

**Figure 5:**
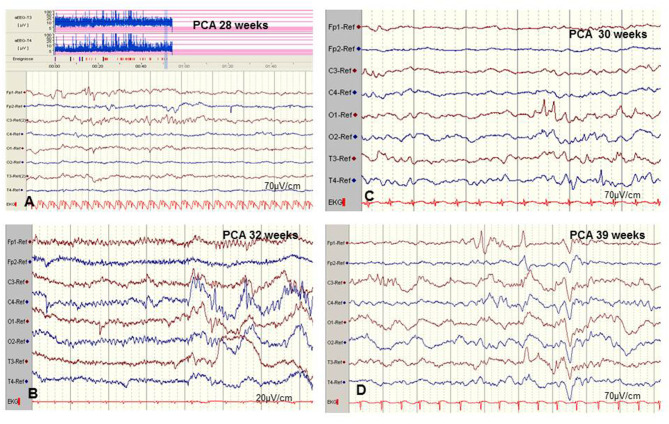
Limits of aEEG monitoring in detection of seizure activity. **(A)** Low-voltage and brief (<10–30 s) epileptic discharges in a preterm of 28 weeks postconceptual age (PCA) not detected by the time-compressed display. **(B)** Pseudosuppression of background due to artifacts of high-frequency oscillatory ventilation. **(C,D)** Physiological paroxysmal transients. **(C)** Sharp theta on the occipital area of prematures (STOP) in a preterm of 30 weeks PCA. **(D)** Frontal sharp theta transients and delta brushes in a term newborn. (Calibration is given in the figure, low-pass 30 Hz, high-pass 0.5 Hz).

However, there are significant methodological limitations of aEEG monitoring. Studies on the sensitivity of aEEG in the detection of seizure activity in pre-terms and critically ill neonates are rare and most often very heterogenous regarding gestational age (GA), electro-clinical diagnosis, and technical methods, which vary from 2–4 (cerebral function monitoring, CFM) to 8–10 active electrodes (aEEG) (48, 49). Furthermore, results on inter-observer reliability in interpreting neonatal aEEG are inconsistent (49–51). Well-known limits of aEEG monitoring include a low sensitivity for low voltage, as well as brief (<10–30 s) epileptic discharges (Figure 5A) and age-related maturational pattern (physiological paroxysmal transients) not being detected (Figures 5C,D).

Moreover, detection of short ictal suppression or transient suppression in response to sedative or analgesic medication (29) is impossible without additional multichannel EEG recording. An example of “pseudosuppression” of background due to artifacts of high-frequency oscillatory ventilation is shown in Figure 5B. Furthermore, reported experience in seizure detection in extremely pre-term infants (GA 24–28 weeks) is limited (18, 27, 35, 52). A systematic review reported a sensitivity of 76% and a median specificity of 85% of aEEG for the detection of seizure activity in neonates (53). In terms of these methodical limitations, aEEG monitoring is recommended as a useful bedside method for continuous and long-term monitoring in neonates at risk for seizures, but as a complementary tool to multichannel EEG [American Clinical Neurophysiology Society's Guidelines on EEG Monitoring in Neonates; (22, 32)].

### Continuous EEG Monitoring in Electrographic-Only Seizures

Data on “electrographic-only” seizures, recently recommended as a defined seizure type of the neonate by the ILAE Task Force on Neonatal Seizures (8), are limited and include heterogeneous study groups in terms of definition, age distribution, and technical conditions (18, 27, 33, 34, 42, 54). Electrographic-only seizures that are characterized by a “repetitive and evolving pattern with a minimum 2 μV voltage and duration of at least 10 s” (32) are commonly described in both pre-term and term infants with acute symptomatic seizures and encephalopathies such as hypoxic-ischemic or infectious encephalopathies (8, 55), as well as structural and genetic neonatal-onset epileptic encephalopathies (8, 33, 42).

In term infants with HIE receiving therapeutic hypothermia (TH), comprehensive multicentre studies have confirmed the high risk of electrographic-only seizures. The fraction of electrographic-only seizures has been found as high as 50–80% of the whole seizure burden (8, 55). During TH (72 h), Nash et al. (55) detected seizures in 34% and status epilepticus in 10% of term infants with HIE, and more than 50% of these seizures were electrographic-only. In term neonates with moderate HIE, TH has been shown to reduce the degree of electrographic seizure burden (5, 56, 57); however, more than 50% of infants with moderate HIE developed seizures during TH and after its cessation [>72 h; (5, 28, 29)]

Together, aEEG has been shown to enable the detection of recurrent electrographic-only seizures and neonatal status epilepticus with high sensitivity in selected populations (18, 32, 33). However, the detection rate is significantly limited in the case of low-amplitude epileptic discharges and repetitive seizure activity of short duration (<10 s) (3, 58, 59); thus, a combination of aEEG and multichannel EEG is required (22, 32).

## EEG Monitoring in Neonatal-Onset Epileptic Encephalopathies

### EEG Monitoring in Perinatal HIE

In particular in term neonates with HIE, aEEG combined with multichannel EEG has been proposed as a reliable diagnostic and prognostic tool (22, 60, 61). Based on large international studies (4, 62), the significance of background suppression and burst suppression, which represent previously established prognostic EEG patterns in perinatal HIE, needs to be re-evaluated in the era of TH, in part: Initial burst suppression was not associated with death or severe disability in about 40% of the affected infants (4). Thus, initial burst suppression does not exclude normal neurological development, and sequential EEG recordings during the postnatal interval of 36–48 h are recommended for estimating prognostic aspects and long-term outcomes (4, 60–62). Based on comprehensive analyses of the prognostic value of aEEG criteria in HIE it remains unchanged, that the rapid improvement of an initial suppression [<24 h; (16, 26, 62)] and the early development of sleep-wake cycling (<36 h) are associated with a favorable neurodevelopmental outcome (55, 60).

The *prognostic significance of prolonged epileptic discharges* and high seizure burden in neonates with HIE is well-described. In term neonates with HIE and TH, a high frequency of electrographic seizures has been strongly associated with severe hypoxic and ischemic cMRI lesions and abnormal development (17). Using cEEG for at least 72 h in term neonates with moderate or severe HIE, Weeke et al. (35) found a significant relationship between the total seizure burden and neurodevelopmental outcomes at the age of 24 months, with a PPV of 78% and an NPV of 71%. Similarly, unfavorable neurodevelopmental outcomes in infants with HIE and prolonged electrographic-only seizure activity have been observed by others (16, 17, 63, 64). Using multivariate analysis, Fitzgerald et al. (17) identified the prognostic significance of prolonged seizure exposure in term infants with HIE (*N* = 93) and proposed a high frequency of electrographic seizures (OR 5.2, 95% CI 1.3–21.2, *p* = 0.02) and moderate or severe slowing of background activity (OR 8.3, 95% CI 1.6–43.9, *p* = 0.01) as predictive parameters for abnormal motor development, and a high electrographic seizure frequency for deficiencies of speech development (OR 4.2, 95% CI 1.1–15.9, *p* = 0.04). Repetitive electrographic seizures or status epilepticus pattern during the first 24 h after rewarming but not the first 24 h after cooling were associated with post-neonatal epilepsy in patients with moderate or severe HIE at the age of 12 months (65). Increased hippocampal apoptosis and elevated IL-8 and inflammatory cytokine levels may lower the seizure threshold during the acute period of HIE and increase the risk of post-neonatal epilepsy [6–22%; (57)]. However, comparison of different predictors of outcomes in neonates with HIE and TH showed a higher specificity of neonatal cMRI than EEG/aEEG and neonatal electrographic seizure burden (61–65). The strength of EEG/aEEG is its high overall availability during the early postnatal period compared to cMRI (56, 58, 59), its high sensitivity to identify electrographic seizures and abnormal background activity, and to monitor therapeutic management.

### EEG Monitoring in Structural and Metabolic Neonatal-Onset EE

In general, abnormal EEG findings in structural and metabolic neonatal-onset EE are not specific; however, they could point to the differential diagnoses of structural brain anomalies or inherited metabolic diseases, and direct specific investigations. Suppression burst pattern in neonates with refractory seizures may indicate severe brain malformation as well as metabolic diseases, e.g., sulphite oxidase deficiency, molybdenum cofactor deficiency, pyridoxine-dependent epilepsy, non-ketotic hyperglycinemia (Figures 2A,B), mitochondrial cytochrome c oxidase deficiency or pyruvate dehydrogenase complex deficiency (Figures 2C,D), and lipoic acid synthetase deficiency (66). In pyridoxine-dependent epilepsy, continuous high-voltage rhythmic delta slow waves have been reported as a characteristic EEG pattern (60). Appropriate treatments other than anticonvulsive drugs may improve the course of these rare EE. Concerning structural neonatal-onset EE, rhythmic and high-amplitude alpha or beta activity in all cortical regions may indicate the so-called lissencephaly-pachygyria spectrum based on disturbances of neuronal migration (67).

### Electro-Clinical Findings in Genetic Neonatal-Onset EE

In neonatal-onset EE, a genetic etiology is increasingly identified. In a prospective cohort of neonates with seizures enrolled in the multicentre US Neonatal Seizure Registry (11), neonatal EE was found in 13% of patients (79/611), and 83% had a genetic etiology, with *KCNQ2* variants the most common. Using targeted gene sequencing, Na et al. (12) recently identified pathological variants in 42.9% (30/70) of infants with neonatal-onset EE, with the most common pathogenic variants detected in the *KCNQ2, STXBP1*, and *CDKL5* genes (20/30, 66.7%). Similarly, Lee et al. (10) identified 31 patients (64.4%) with a genetic etiology among a total of 48 neonates with EE with burst suppression, with the most commonly diagnosed pathogenic variants in the *STXBP1* (27.1%), *KCNQ2* (10.4%), and *SCN2A* genes (10.4%).

To optimize short- and long-term management and targeted treatment options (68–71), a better understanding and characterization of the electro-clinical phenotypes of genetic neonatal-onset EE is increasingly important and highly challenging. At present, no electrographic pattern specific for a defined genetic etiology have been identified in genetic neonatal-onset EE. However, supported by the recommendations of the American Clinical Neurophysiology Society (22), the increasingly common use of EEG monitoring in at-risk neonates might lead to increased experience in the characterization of the electro-clinical phenotypes of neonatal-onset EE (71–74). Rare neonatal-onset, severe EE such as Ohtahara syndrome [OS; syn. early infantile EE (EIEE)], early myoclonic encephalopathy (EME), and vitamin-dependent epilepsies are well-characterized electro-clinically, and increasingly genetically (10, 12, 13). The hallmark of OS is an unresponsive and invariant burst suppression pattern with bursts of high-amplitude spikes and polyspikes alternating with short periods of suppression in a regular pattern. Burst suppression in EME occurs predominantly during sleep and might be not continuous. As a monogenic etiology of these severe neonatal-onset EE, as well as EE other than EIEE/EME (10), is increasingly confirmed, elucidating novel underlying disease mechanisms (12, 13, 68, 74) as well as prognostic or therapeutic implications (68, 73, 74), a future adaptation of the presently used electro-clinical classification has been proposed (8, 9, 71).

The following section includes examples of EEG findings in rare genetic neonatal-onset epilepsies with monogenic variants without significant structural brain anomalies. This group of EE etiologically includes channelopathies (e.g., *KCNQ2/3, KCNT1, SCN2A*), cell signaling disorders including developmental transcription factor and RNA processing disorders (e.g., *FOXG1, GNAO1, BRAT1, CDKL5*), synaptopathies and synaptic transmission disorders (*STXBP1, DNM1, SPTAN1*) and mitochondrial disorders (e.g., *PDHA1, PDHB, PDHX, POLG1*) (11, 71, 75). Even if pathognomonic EEG findings are missing - except for typical EEG patterns associated with individual variants (e.g., *KCNQ2, KCNT1*)—analysis of EEG examinations in these rare genetic neonatal-onset EE will lead to a better understanding of the clinical and EEG phenotypes.

#### EEG Characteristics in Neonatal-Onset Channelopathies

##### Neonatal-Onset KCNQ2 Encephalopathy

Mutations in the *KCNQ2* gene encoding the voltage-gated potassium channel K_V_ 7.2 are well-known as the genetic cause of self-limited familial neonatal seizures. However, *de novo KCNQ2* variants were also recognized as causative mutations of EOEE of varying severity (76, 77). The hypothesis is that mutations with a dominant negative effect may explain the more severe functional deficit associated with severe encephalopathy, but the exact mechanisms are not fully understood (13, 76, 78). The reported clinical features of *KCNQ2* encephalopathy include onset within the first week of life, seizures of high frequency with a prominent focal tonic component and autonomic symptoms, and signs of progressive encephalopathy with hypotonia, decreasing vigilance, and reactivity. Seizures are often accompanied by apnoea and, rarely, prolonged bradycardia increasing the risk of sudden unexpected death in epilepsy [SUDEP; (75, 76)]. Associated unspecific neuroimaging features have been reported (66, 77, 79).

*Electrographic characteristics* are background suppression or suppression burst as well as multifocal epileptiform discharges (76, 77, 79). Ictal EEG has revealed unilateral low-voltage fast activity followed by repetitive focal series of spikes and waves (77). Postictally, prolonged and diffuse depression of amplitudes is typically present.

From early childhood, the frequency and severity of seizures improve, but severe neurodevelopmental and intellectual disability is present in almost all patients (76, 77). Sodium-channel blockers such as phenytoin and carbamazepine are recommended as possible precision medicine treatments for *KCNQ2*-related encephalopathies (74). In 11 patients with loss-of-function *KCNQ2* variants, early treatment with ezogabine was found to improve refractory seizure activity without severe side effects (76).

##### KCNT1-Associated Epileptic Encephalopathy

*KCNT1* encodes a voltage-dependent sodium-activated potassium channel subunit mediating slow hyperpolarization. Gain-of-function mutations leading to increased current amplitude in the sodium-activated potassium channel are increasingly recognized as disease-causing variants in epilepsy of infancy with migrating focal seizures (EIMFS) and West syndrome, but neonatal-onset EE is also commonly reported (68, 80–82). More than 50% of known variants are *de novo* (81). Interactions with complex neuronal protein networks such as the fragile-X mental retardation protein may explain the various comorbidities and progressive encephalopathy (82). Commonly reported clinical features of *KCNT1* encephalopathy in the initial phase of the epilepsy represent focal, alternating motor seizures (tonic > clonic), which typically present in the oro-facial region (64–83%), including lateral deviation of the eyes, as well as myoclonic limb jerks and spasms. Autonomic symptoms have been reported in 17–100% of patients presenting apnoeic spells, instability of body temperature, vomiting, or deep breathing (80–82). The second and third phases of the epilepsy are characterized by clusters of focal seizures several times a day, hypotonia and somnolence, and later on, improvement of seizure frequency but severe developmental disability (80, 81).

**Figure 6:**
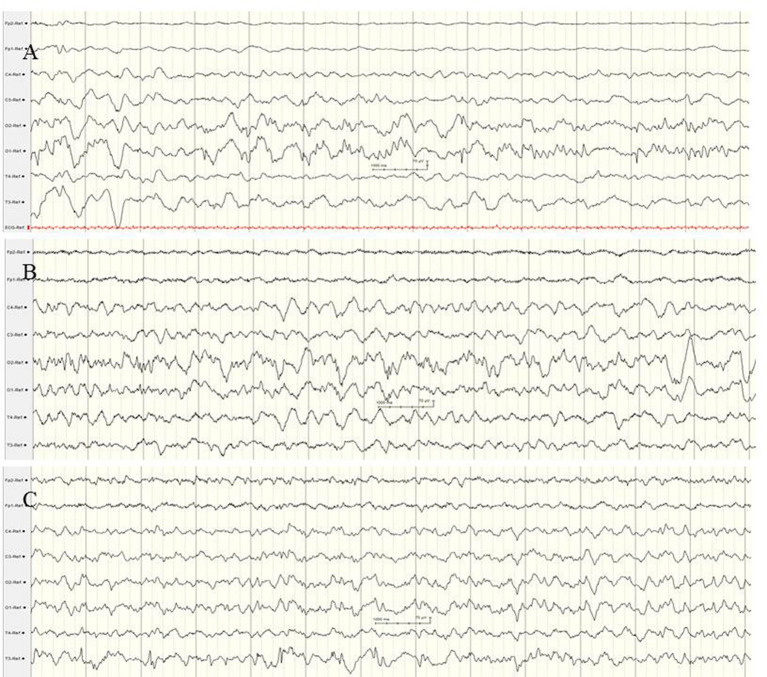
EEG findings in a female newborn with KCNT1-associated epileptic encephalopathy. The girl presented orofacial myoclonic seizures, bulbus deviation, and apnoeic spells. Interictal **(A)** and ictal **(B,C)** EEG at the age of 5 weeks. **(A)** Diffuse slowing of the background and occipital epileptiform discharges. **(B,C)** Ictal EEG with diffuse slowing and rhythmic low voltage epileptiform discharges over the centro-temporal and occipital regions with alternating laterality. (Calibration is given in the figure, low-pass 30 Hz, high-pass 0.5 Hz).

*Typical electrographic findings* have been shown. These were diffuse slowing of the interictal background followed by focal epileptiform discharges with alternating laterality and progressive diffuse slowing, as well as ictal migrating patterns (80–82) (Figure 6). Electrographic-only seizures with focal series of rhythmic alpha or theta and varying ictal onset have been described. Of note, a suppression burst EEG pattern during the first weeks of life has been reported in several patients (68, 80–82), consistent with the electro-clinical phenotype of OS. A recent retrospective study including 19 patients with EIMFS and *KCNT1* mutations analyzed EEG findings after the age of 2.5 years (80). During the third phase, interictal EEGs were continuously abnormal with irregular and slow background activity (100%) with temporal or frontal paroxysmal abnormalities (57%) that were mainly recorded during sleep (80). Ictal EEGs showed focal epileptic discharges also involving mainly frontal and temporal areas.

Quinidine has been suggested as a possible precision medicine treatment (82–84). Severe neurodevelopmental regression and cognitive disability have been reported in all patients, accompanied by dystonic and choreoathetotic movement disorders [23%; (80, 81)]. Associated cardiac anomalies may contribute to the increased SUDEP prevalence in *KCNT1*-associated EE (80).

##### SCN-Associated Early-Onset Epileptic Encephalopathies

Early-onset epilepsies caused by genetic variants in the voltage-gated sodium channel (SCN) gene family are rare and associated with variable phenotypes such as Dravet syndrome, Dravet-like phenotypes, and EIMFS, most often due to missense and protein truncation variants as well as deletions. Additionally, deletions of 2q including *SCN1A, SCN2A*, and *SCN3A* have been associated with developmental delay, dysmorphic features, behavioral problems and psychiatric disorders (85, 86). Genotype–phenotype studies of patients with mutations in the *SCN2A* gene encoding the voltage-gated sodium channel Nav1.2 showed early onset of *SCN2A*-associated EE (<3 months of age) in around 35% of patients (70), and this early-onset group presented with missense mutations that led to a gain of function of the Nav1.2. channel. The degree of gain-of-function was related to the epilepsy phenotype and treatment response to phenytoin (70). The well-characterized clinical phenotype is benign familial neonatal-infantile epilepsy, but *de novo SCN2A* missense variants also cause severe phenotypes, including refractory, neonatal-onset EE. Clinically, neonates with *SCN2A*-associated EE usually reveal focal tonic seizures and apnoea. Treatment response to phenytoin is typically reported (70, 87). *Electrographic findings* have been suppression burst patterns or multifocal epileptic discharges associated with abnormal slowing and suppression of background.

Neuro-developmental outcomes were usually severely impaired (70, 87).

**Figure 7:**
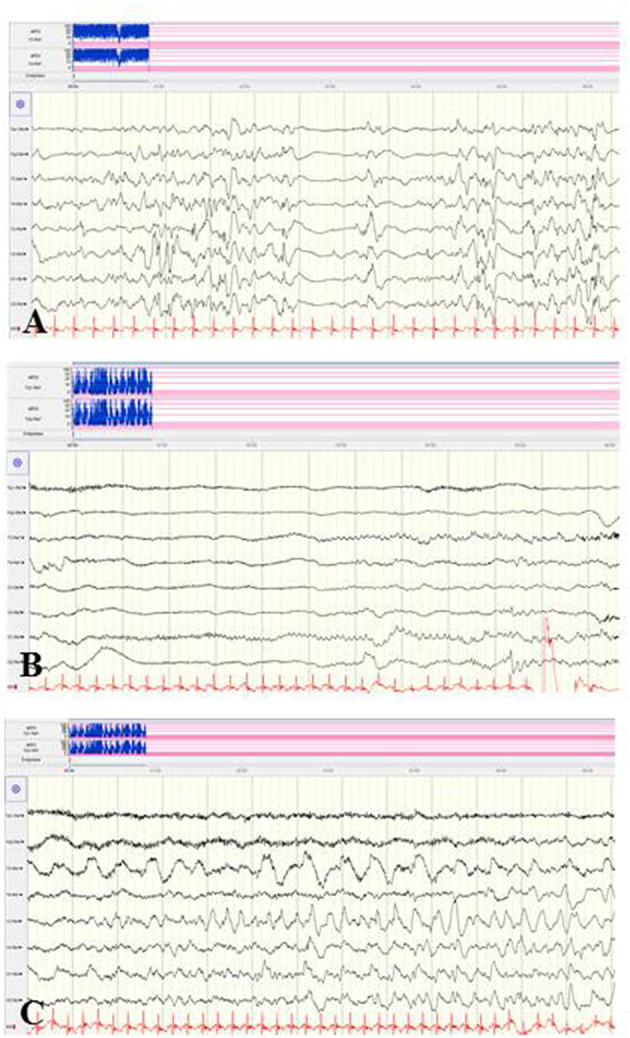
EEG findings of a female neonate with seizure onset in the first day of life caused by a *de novo* duplication of the SCN gene cluster of chromosome 2q24. **(A)** Suppression burst during asleep and awake periods. **(B,C)** Ictal pattern was initial suppression followed by focal epileptic discharges and continuously suppressed background. (Calibration 100 μV/cm, low-pass 30 Hz, high-pass 0.5 Hz).

The rarely described deletion of the whole sodium channel gene cluster (*SCN3A, SCN2A, SCN1A, SCN9A*, and *SCN7A*) has been associated with refractory infantile epilepsy of the EIMFS phenotype (88). In contrast, few clinical reports on epilepsies caused by duplications in the SCN cluster are available (89–93). Figure 7 shows the EEG findings of a female neonate with seizure onset on the first day of life caused by a *de novo* duplication of the SCN gene cluster of chromosome 2q24, including triplication of the *SCN2A* gene. Focal clonic and focal tonic seizures and ictal apnoeic spells were accompanied by a suppression burst and, from the second week of life, prolonged focal epileptic discharges and suppressed background.

##### CACNA1A-Associated Neonatal-Onset Epileptic Encephalopathies

**Figure 8:**
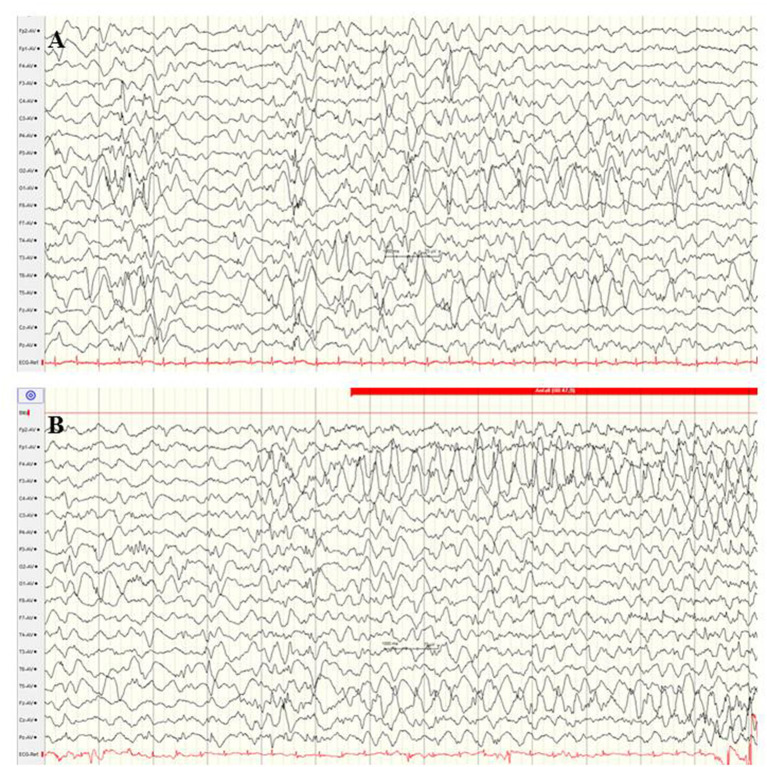
EEG findings of a male neonate with onset of apnoeic spells and focal seizures at the age of 4 weeks. A novel compound heterozygosity for two inherited frameshift mutations in the CACNA1A gene was identified. **(A)** Interictal EEG shows suppression burst during both awake and asleep states. **(B)** Ictal EEG pattern was 2–3 Hz monomorphic, high-voltage epileptic discharge activity. (Calibration 100 μV/cm, low-pass 30 Hz, high-pass 0.5 Hz).

Mutations in the *CACNA1A* gene, which encodes the alpha-1 subunit of a voltage-gated P/Q-type calcium channel, rarely cause variable phenotypes of EOEE (75, 94, 95). Among 531 individuals with a variable spectrum of unsolved EE, six infants with *CACNA1A* mutations (*de novo* 3/5) were identified (75). Five of these infants presented their first clinical seizures during the neonatal period, including myoclonic and tonic seizure types. Exclusively compound heterozygous mutations in the *CACNA1A* gene have been associated with severe EE with early infantile onset, severe muscular hypotonia, and progressive cerebral, cerebellar and optic nerve atrophy (94). Figure 8 shows the EEG findings of a male neonate with onset of apnoeic spells and focal seizures at the age of 4 weeks, in whom a novel compound heterozygosity for two inherited frameshift mutations in the *CACNA1A* gene was identified [maternal: c.2602delG p. (Ala868Profs^*^24), paternal: c.5476delC p. (His1826Thrfs^*^3)]. He developed a rapid progressive EE with refractory focal seizures and apnoea, severe muscular hypotonia, optic atrophy, and lethal course at the age of 2.5 years. EEG showed suppression burst during both awake and asleep states (Figure 8). During apnoeic spells as well as postictally, prolonged and severe suppression was present. Under anticonvulsive treatment, the EEG remained severely abnormal with generalized slowing and 2–3 Hz monomorphic, high-voltage activity and diffuse sharp and spike waves (Figure 8). A similar EEG pattern was observed in two sisters with severe EE associated with a compound heterozygous missense mutation in exon 27 (c.4315T > A) and exon 3 (del c.472_478delGCCTTCC) (94).

Interestingly, this suppression pattern is in line with recent experimental reports. Studies on adult homozygous *Cacna1a*^S218L^ mice functionally characterized by gain-of-function of voltage-gated CaV2.1 Ca^2+^ channels (96) observed multiple spontaneous tonic-clonic seizures and fatal apnoea in these transgenic mice, accompanied by ictal and postictal suppression of cortical neuronal activity (96). Mechanisms of seizure-related apnoea in *CACNA1A* mutations, which implicate a high risk of death and SUDEP, are not fully understood. The application of the NMDA receptor antagonists MK-801 and memantine-induced stabilizing effects on brain stem function in adult transgenic *Cacna1a*^S218L^ mice (97).

#### EEG Characteristics in Neonatal-Onset EE Based on Cell Signaling Disorders

##### GNAO1-Associated Early-Onset Epileptic Encephalopathy

**Figure 9:**
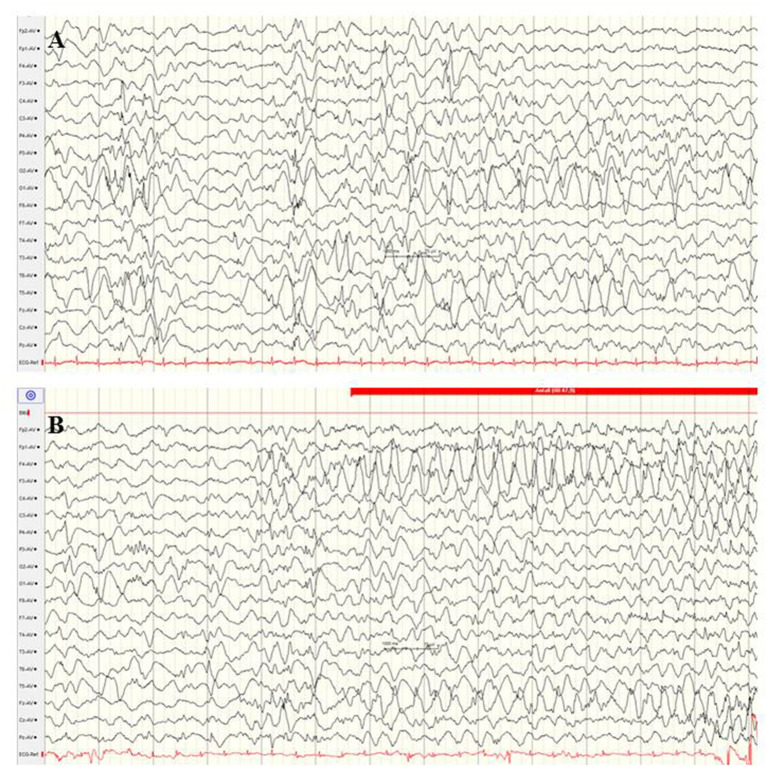
EEG in a female newborn with GNAO1 mutation presenting multifocal tonic seizures and apathy from the 2nd week of life. **(A)** Interictal EEG at the age of 3 weeks with suppression burst. **(B)** Ictal EEG reveals monomorphic, high-amplitude epileptic discharges over the fronto-central regions and diffuse slowing (Calibration 100 μV/cm, low-pass 30 Hz, high-pass 0.5 Hz).

The *GNAO1* gene, encoding the G-protein subunit of heterotrimeric guanine nucleotide-binding proteins, is involved in regulating neuronal excitability and neurotransmission. Additionally, the G-protein–cAMP pathway axis represents a key contributor to the pathophysiology of dystonia and chorea (98). At present, observations about *GNAO1*-associated encephalopathy are limited, and around one-third of the reported patients showed neonatal-onset EE (66). These neonatal seizures are described as focal motor seizures with high seizure frequency and associated critical bradycardia (99). Several patients showed cerebral atrophy, delayed myelination, and thin corpus callosum. *Electrographic findings* were a suppression burst or abnormal background associated with multifocal sharp waves (Figure 9). Severe dystonic movement disorders usually persist, as well as neurodevelopmental delay (99).

#### EEG Characteristics in Neonatal-Onset EE Based on Disorders of Synaptic Transmission

##### STXBP1-Associated Neonatal-Onset Epileptic Encephalopathy

**Figure 10:**
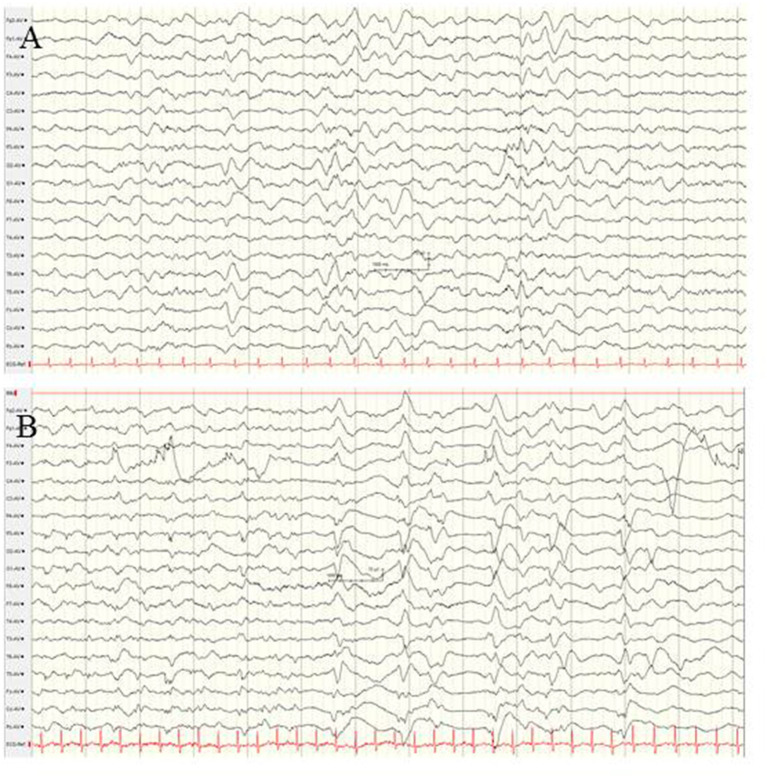
EEG findings in a female neonate with STXBP1-associated EE at the age of 4 weeks. **(A)** Interictal EEG showing diffuse slowing, and low-voltage fast activity and occipital spikes. **(B)** Ictal EEG reveals periodic patterns and suppression burst. (Calibration is given in the figure, low-pass 30 Hz, high-pass 0.5 Hz).

The syntaxin-binding protein 1 (*STXBP1*) gene, which encodes for a membrane-trafficking protein that modifies synaptic vesicle function, is crucially involved in presynaptic vesicular fusion reaction, neurotransmitter secretion, and maintenance of GABAergic and glutamatergic neuronal synapses (100, 101). *De novo STXBP1* mutations are among the most common causes of neonatal-onset genetic EE or OS (13, 101–103). Beyond the neonatal period, *STXPB1* mutations have been found in infants with West syndrome, Dravet syndrome, non-syndromic refractory epilepsy and intellectual disability, atypical Rett syndrome and autism (103–106). The semiology of seizures in neonates with *STXBP1* mutations has been described as focal seizures and epileptic spasms (102, 103). In 7/14 patients with *STXBP1*-associated encephalopathy presenting with EE within the first 4 weeks of life (102), semiology has been heterogeneous, including focal and bilateral clonic convulsions, spasms, and tonic seizures. The *electrographic findings* at seizure onset were suppression burst, diffuse slowing, and focal epileptic discharges [(102); Figure 10]. Improvement of seizure frequency during early childhood has been reported. Persistent profound developmental impairment and ataxic and dystonic movement disorders determine long-term morbidity (101, 107).

## Summary

Neonatal EEG remains a highly sensitive neurophysiological method for detecting focal and diffuse cerebral dysfunctions and electrographic seizure activity. Amplitude-integrated EEG is highly recommended as a useful bedside monitoring method in at-risk neonates but only as a complementary tool to multichannel EEG because of methodical limitations. EEG/aEEG is highly sensitive to identify electrographic seizures and abnormal background patterns in neonates at risk for prolonged and/or electrographic-only seizures including newborns with HI, metabolic, or genetic encephalopathies; however, its prognostic significance is low in all etiological groups. In neonates with HIE and TH, the predictive significance of EEG/aEEG is significantly lower compared to cMRI. Electrographic patterns specific for a genetic variant have not been characterized in neonatal-onset EE; however, extending the present knowledge about EEG monitoring data may increase awareness for specific etiological clarification of neonatal-onset EE.

## Author Contributions

RT wrote the paper and performed all the work.

## Conflict of Interest

The author declares that the research was conducted in the absence of any commercial or financial relationships that could be construed as a potential conflict of interest.

## Abbreviations

aEEG: amplitude-integrated electroencephalography
cEEG: conventional electroencephalography
EIEE: early infantile epileptic encephalopathy
EME: early myoclonic encephalopathy
EOEE: early-onset epileptic encephalopathy
GA: gestational age
HIE: hypoxic-ischemic encephalopathy
PCA: post-conceptual age
OS: Ohtahara syndrome
TH: therapeutic hypothermia.
